# Detection of Pseudocyst Forms of *Trichomonas muris* in Rodents from Iran

**Published:** 2018-05

**Authors:** Zabiholah ZAREI, Mehdi MOHEBALI, Khadijeh KHANALIHA, Eshrat Beigom KIA, Afsaneh MOTEVALLI HAGHI, Zahra HEIDARI, Tahereh REZAEIAN, Mostafa REZAEIAN

**Affiliations:** 1. Dept. of Medical Parasitology and Mycology, School of Public Health, Tehran University of Medical Sciences, Tehran, Iran; 2. Center for Research on Endemic Parasites of Iran (CREPI), Tehran University of Medical Sciences, Tehran, Iran; 3. Research Center of Pediatric Infectious Diseases, Institute of Immunology and Infectious Diseases, Iran University of Medical Sciences, Tehran, Iran

**Keywords:** *Trichomonas muris*, Pseudocyst, Rodents, Iran

## Abstract

**Background:**

*Trichomonas muris* is one of the most common protozoa diagnosed in rodents. The trichomonads are generally described as presenting only trophozoite form while pseudocyst is another morphological form of trichomonads identified among gastrointestinal and genitourinary trichomonads. We identified and described different shapes of *T. muris* pseudocysts and trophozoite in stool samples were collected from rodents including *Merinos persicus*, *Mus musculus* and *Cricetulus migratorius*.

**Methods:**

In this cross-sectional study, stool samples from 204 trapped rodents were collected from Meshkin Shahr during Mar to Dec 2014. Samples were preserved in formalin 10% and PVA solution and transferred to Department of Medical Protozoology and Mycology, School of Public Health, Tehran University of Medical Sciences. Formalin-ether concentration method was used for the samples. The slides were stained with tri-chrome staining method and observed under light microscope.

**Results:**

The trophozoites were classified as *T. muris* based on size (18 to 24 μm), presence of three anterior flagella, recurrent flagellum, undulating membrane, and axostyle in direct examination and stained slides with trichrome staining method. Fifty-five out of 204 (27%) rodents were infected with *T. muris* in which 51(25%) samples pseudocysts form were observed. The spherical bodies of pseudocyst with almost 8 μm size, contained internalized flagella, an undulating membrane with recurrent flagellum, axostyle, and costa were seen. The pseudocysts were more prevalent than trophozoite form and pseudocysts were found with different shapes in this study.

**Conclusion:**

*T. muris* pseudocysts were found in stool samples of caught rodents for the first time in northwestern Iran.

## Introduction

*Trichomonas muris* is one of the most common protozoa diagnosed in rodents (1, 2). *T. muris* trophozoites are found in the cecum and large intestine of different rodent species, including mice, rats, and hamsters (3). The trichomonads are generally identified as showing only the trophozoite form, although some species present the pseudocyst form (4, 5). The transmission happened by ingestion of pseudocyst from feces of infected mouse. *T. muris* multiply in large intestine without invading to intestine. Although *T. muris* almost assumed nonpathogenic protozoa (3), diarrhea, and anorexia have been reported in some studies (6).

Pseudocyst formation is a morphological transfiguration of the trophozoite into close-packed, non-motile form, without true cyst wall. This form has been reported previously in some trichomonads that live in gastrointestinal system (1, 7). In the recent years, pseudocyst was described among genitourinary trichomonads (4).

There are a few reports about parasitic infection of rodents in Meshkin Shahr area, northwestern of Iran (8, 9).

In this study, we identified and described the different shapes of *T. muris* pseudocyst for the first time in 204 stool samples collected from rodents including Merinos *Persicus*, *Mus musculus* and grey hamster from Meshkin Shahr area, northwestern of Iran.

## Material and Methods

### Sample collection

In this cross-sectional study, stool samples from 204 trapped rodents including *M. persicus* (no.117), *Mus musculus* (no.63) and *Cricetulus migratorius* (gray hamster) (no.24) were collected by Sherman method, live animal traps from Meshkin Shahr area, northwest of Iran from Mar to Dec of 2014 (10). The study was done in compliance with current national laws and regulations. Samples were preserved in formalin 10% and PVA solution and transferred to Department of Medical Protozoology and Mycology, School of Public Health, Tehran University of Medical Sciences, Iran. Formalin-ether concentration method used for the sample preparation and all of samples examined with light microscope with 100X and 400 magnifications. Wet smears were prepared for detecting of trophozoite form of *T. muris.*

### Trichrome staining method

Some positive *T. muris* slides were stained with trichrome staining method (11). The slides were mounted using Canada balsam and observed under 1000 X final magnification under light microscope.

Analysis detection of *T. muris* pseudocyst was made based on morphological characteristic for pseudocyst (12). Analysis was performed using Excel 2007.

### Ethical approval

This study was approved by the Ethics Committee of Tehran University of Medical Sciences (Ethic no. 25287) in accordance with Helsinki Declaration and guidelines.

## Results

From 204 of the rodents were trapped, 127(62.3%) of them were male and 77(37.7%) were female. In general 55 out of 204 (27%) rodents were infected with *T. muris* including *Merinos persicus* 40/117 (34.2%), *Mus musculus* 7/63 (11.1%) and *C. migratorius* 8/24 (33.3%), in which 51/204 (25%) samples pseudocysts form were observed. The most common frequency of *T. muris* was found in *Merinos persicus* including 21/69 (30.4%) male and 19/48 (39.6%) female followed by *C. migratorius* including 8/16 (50%) male and then *Mus musculus* including 5/42 (11.9%) male and 2/21 (9.5%) female. Frequency of *T. muris* infection in male rodents 34/127 (26.8%) was almost equal with female rodents 21/77 (27.3%) (Table 1).

**Table 1:** Frequency of Intestinal *T. muris* among 204 caught rodents from Meshkin Shahr area, Ardabil province in 2014

***Parasite***	***Merinos persicus***		***Mus musculus***		***Cricetulus migratorius***		***Total n(%)***		**Total**
	**Male**	**Female**	**Male**	**Female**	**Male**	**Fmale**	**Male**	**Female**	
*T. muris* trophozoite and pseudcyst	21(30.4)	19(39.6)	5(11.9)	2(9.5)	8(50)	0(0)	34(26.8)	21(27.3)	55(27)
Negative *T. muris* samples	48(69.6)	29(60.4)	37(88.1)	19(90.5)	8(50)	8(100)	93 (73.2)	56(72.7)	149(73)
Total	69	48	42	21	16	8	127	77	204

**Fig. 1:**
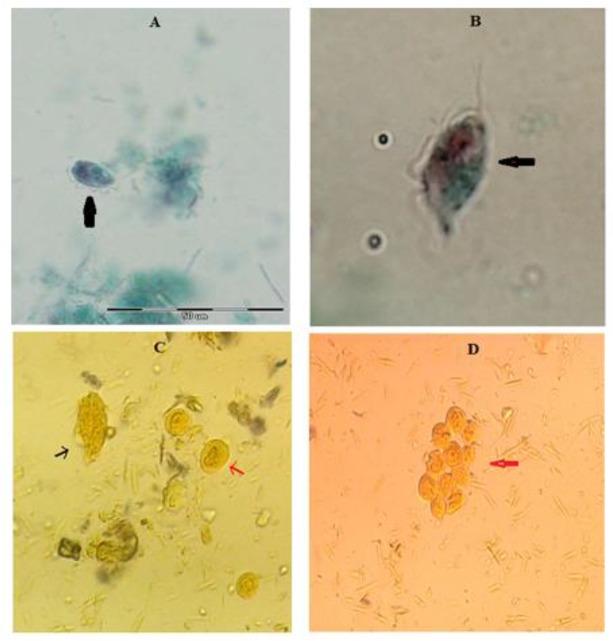
A, B: Trophozoite of *T. muris* under light microscope with 1000X magnification (trichrome staining); C: Trophozoite (black arrow) and pseudocyst (red arrow) form of *T. muris* in rodents stool samples (wet mount); D: pseudocyst form of *T. muris* in rodents stool samples, pay attention to spherical pseudocysts and flat, irregular pseudocysts shapes (original pictures)

The most common protozoa in stool samples were *T. muris*. Trophozoites were classified as *T. muris* on the basis of size (18 to 24 × 12 to 14 μm), presence of three anterior flagella, recurrent flagellum, undulating membrane, and axostyle in direct examination and stained slides with tri-chrome staining method (Fig. 1: A, B).

The spherical bodies contained internalized flagella, an undulating membrane with recurrent flagellum, axostyle, and costa was seen (Fig. 1: C, D). Identification of the bodies as pseudocysts of *T. muris* was made after observing characteristic morphology like rounded cells with almost 8 μm in diameters without true cell wall and without external flagella.

Finally, confirmation of pseudocysts in fresh samples was made according to characteristic morphology (12). The pseudocysts were visible in concentrated smears and in stained smear with trichrome staining method.

The pseudocyst form was more prevalent than trophozoite and *T. muris* pseudocysts in different shapes and numbers were seen in rodents stool samples (Fig. 1: D).

Some pseudocysts were dark and flat with irregular, angled shape that larger than others and when they are stained with eosin they didn’t get color, but some pseudocysts shape were spherical and bright its almost 8 μm and get color of eosin easily.

## Discussion

*T. muris* is most common protozoa in rodents’ infection (1, 2). Pseudocyst form of trichomonads was recognized among gastrointestinal and genitourinary trichomonads and the transmission of *T. muris* occurred by ingestion of pseudocyst from infected mouse (1, 4, 6); therefore, study on the different shape of parasite has medical and veterinary importance.

“*T. muris* is not believed to be pathogen and uses as a contamination biomarker in protozoa-free rodent colonies because of its low minimal infective dose” (3). The minimum dose of contamination is almost 5 pseudocysts; therefore, *T. muris* free rodents are helpful index in experimental studies (13).

Three different forms of the *T. muris* were described: trophozoite form that proliferate by binary fusion; an intermediate form that has oval to pear shape of the trophozoite and is active phagocytic cell but its flagella has internalized; and the pseudocyst or infective form of parasite. *T. muris* does not have the ability to make a true cyst (14). Pseudocyst formation occurred under experimental stress conditions such as pH changes or nutrients consumption, it is actually a defense mechanism of the parasite; and it happens in natural culture medium (4, 15).

In an ultra-structural study of *T. muris* pseudocyst that carried out, the flagella are placed in endocytic vacuoles in beating situation, the axostyle and the costa do not change in integrity but show curved shape, the mitotic process happened within pseudocysts but is different from which reported for trophozoite, and the pseudocysts formation is convertible if the cells are moved to fresh medium (15)

*T. muris* pseudocyst does not have true wall. It has trilaminar wall consisting two radio dense layers that enclosed by a radiolucent layer in electron microscopy studies (15).

Transmission of *T. muris* pseudocyst to mice has been described. After 6 wk infected fecal exposure to free contaminated mice, trophozoites were found in association with pseudocysts in the lumen of large intestinal in the mice. Pseudocyst transformation occurred when trophozoite moving through colon and the ratio of trophozoites to pseudocysts ranged from approximately 6:1 in the cecum to 1:1 in the colon (3). In another study, variable numbers of pseudocysts of *T. muris* were excreted in their feces from female golden hamsters made infection in newborn hamster’s ceca with *T. muris* after 7 d (1).

In present study, numerous pseudocysts and some trophozoites were seen in stool samples from rodents and pseudocyst form of *T. muris* are more frequent than trophozoite form that is consistent with another study (3).

Almost 55% of the *T. foetus* were in pseudocyst form in each preputial sample from seven infected cows, whereas 25% of *T. foetus* were in trophozoite form and pseudocysts were more frequently found than the trophozoite form of parasites in each sample (12).

Result of present study showed different shapes of pseudocysts that some of them were large, dark with irregular, angled shape and when they are stained with eosin they did not get color and them while some pseudocysts shape were spherical and bright. It is almost 8 μm and gets color of eosin easily. The number of spherical forms gradually decreased in fresh samples in our study. Four different stages (A, B, C, and D) of pseudocysts could be distinguished in fresh feces that stage A is infective form but stage D or aged pseudocyst is not (16). Some changes were showed when pseudocyst become age in appearance: color (from bright to dark), shape from spherical get to flat), size (from 8 increase to 13 μm diameter), motion of internalized undulating membrane (observable in A and B but no noticeable in C and D) and number of stage A pseudocysts declined in the fresh fecal samples but those of stage D did not (16).

Trichomonad pseudocysts represent deteriorative forms (17, 18). Against this subject in an ultra-structural study about *T. muris* demonstrated that pseudocyst formation is a reversible phenomenon and pseudocyst has normal morphology of internal structure and ability of division and it is infective form of parasite. Furthermore pseudocysts were observed as ameboidal or spherical form presenting intense flagella beating inside the cell (14).

Iron has an important role in pseudocyst formation. Iron depletion from culture medium could transform trophozoite of *T. foetus* to pseudocyst and iron had a modulator role in the parasite phenotype (19, 20).

Pseudocyst was also identified among genitourinary trichomonads. Trichomonads had ability to form pseudocyst under natural and unfavorable conditions, the pseudocyst, trophozoite forms are able to adhere to vaginal epithelial cells, and the adhesion rate is higher for pseudocyst (21).

Pseudocysts form of *T. vaginalis* which isolated from cervical neoplasia patients in terms of surface and ultrastructural and biochemical properties were different from those isolated from noncervical neoplasia patients and proposed the possibility role of pseudocysts stage in intensifying of cervical neoplasia (22, 23). The role of cysteine proteinases in pathogenesis of *T. vaginalis* was demonstrated (24). Pseudocyst form plays a role in parasite transmission and may explain transmission of *T. vaginalis* infection by non-sexual route like clothes and water (23).

Inoculation of *T. vaginalis* pseudocysts to induce trichomoniasis was evaluated in mice. *T. vaginalis* pseudocyst adhered to epithelial cells in contact-dependent manner with higher infectivity and invasive effects than the trophozoite form and *T. vaginalis* pseudocyst is an active form that can induce trichomoniasis (25).

In this study, rodents from Meshkin Shahr area were infected with *T. muris*. Pseudocyst form of *T. muris* are more frequent than trophozoite form in each sample and the most common frequency of *T. muris* was found in Merinos persicus. In addition to trophozoite, pseudocysts form was found in rodents stool samples were collected from Meshkin Shahr area.

## Conclusion

Pseudocysts in different shapes identified and described for the first time in rodents stool samples were collected from Meshkin Shahr area, northwestern Iran.

## Ethical considerations

Ethical issues (Including plagiarism, informed consent, misconduct, data fabrication and/or falsification, double publication and/or submission, redundancy, etc.) have been completely observed by the authors.
